# The prevalence of prostate cancer in Pakistan: A systematic review and meta-analysis

**DOI:** 10.1016/j.heliyon.2023.e20350

**Published:** 2023-09-20

**Authors:** Sohail Akhtar, Fazal Hassan, Sadique Ahmad, Mohammed A. El-Affendi, Muhammad Imran Khan

**Affiliations:** a Department of Mathematics and Statistics, The University of Haripur, Haripur, Pakistan; b EIAS: Data Science and Blockchain Laboratory, College of Computer and Information Sciences, Prince Sultan University, Riyadh, 11586, Saudi Arabia; c Department of Industrial Engineering, Hanyang University, Seoul, South Korea

**Keywords:** Cancer, Prostate cancer, Meta-analysis, Pakistan

## Abstract

**Background:**

Prostate cancer is a significant public health issue, ranking as the second most common cancer and the fifth leading cause of cancer-related deaths in men. In Pakistan, the prevalence of prostate cancer varies significantly across published articles. This study aimed to determine the pooled prevalence of prostate cancer and its associated risk factors in Pakistan.

**Methods:**

MEDLINE (via PubMed), Web of Science, Google Scholar, and local databases were searched from inception until March 2023, using key search terms related to the prevalence of prostate cancer. We considered a random-effects meta-analysis to derive the pooled prevalence and relative risks with 95% CIs. Two investigators independently screened articles and performed data extraction and risk of bias analysis. We also conducted meta-regression analysis and stratification to investigate heterogeneity. This study protocol was registered at PROSPERO, number CRD42022376061.

**Results:**

Our meta-analysis incorporated 11 articles with a total sample size of 184,384. The overall pooled prevalence of prostate cancer was 5.20% (95% CI: 3.72–6.90%), with substantial heterogeneity among estimates (I^2^ = 98.5%). The 95% prediction interval of prostate cancer was ranged from 1.74%–10.35%. Subgroup meta-analysis revealed that the highest pooled prevalence of prostate cancer was in Khyber Pakhtunkhwa (8.29%; 95% CI: 6.13–10.74%, n = 1), followed by Punjab (8.09%; 95% CI:7.36–8.86%, n = 3), while the lowest was found in Sindh (3.30%; 95% CI: 2.37–4.38%, n = 5). From 2000 to 2010 to 2011–2023, the prevalence of prostate cancer increased significantly from 3.88% (95% CI: 2.72–5.23%) to 5.80% (95% CI: 3.76–8.24%).

**Conclusions:**

Our meta-analysis provides essential insights into the prevalence of prostate cancer in Pakistan, highlighting the need for continued research and interventions to address this pressing health issue.

## 1 Introduction

Cancer is a primary reason of death globally, accounting for over 10 million deaths in 2020, or roughly one in every six deaths [[1], [2], [3], [4], [5]]. Prostate cancer is the most prevalent cancer among men and the second leading cause of cancer mortality after skin cancer [6]. Worldwide, an estimated 1,414,259 new cases and 375,304 deaths were linked to prostate cancer in 2020 [6]. The prevalence of prostate cancer varies across different countries, with higher rates found in Eastern countries. In 2020, over 1.4 million new cases and 381,000 deaths were reported due to prostate cancer, making it the most commonly diagnosed cancer among men. By 2030, the number of new cases and deaths will increase significantly [7]. Prostate cancer is a primary health concern and a significant economic burden. According to a study, the global financial cost of prostate cancer was estimated to be approximately $12.1 billion in 2010 [8]. The cost of treatment, loss of productivity, and other indirect costs associated with prostate cancer can devastate families and communities. As age increases, there is a correlation between the prevalence and mortality of prostate cancer worldwide, with an average age of 66 years at diagnosis [9]. It is worth noting that African-American men have higher incidence rates compared to white men, with 158.3 new cases per 100,000 men, and their mortality rates are about twice as high as those of White men [10]. Some Asian countries, such as Israel, Lebanon, Kuwait, the United Arab Emirates, Qatar, Oman, and Japan, have a higher incidence of prostate cancer. The incidence rate of prostate cancer is 10.5 in Eastern Asia, 11.2 in Southeast Asia, and 4.5 in South Central Asia, with rising mortality rates [11,12].

Prostate cancer is among the top ten most common cancers and the third most widespread genitourinary cancer among males in Pakistan [13]. The prevalence of prostate cancer significantly varies in the published articles. There is no country-wide survey to estimate the overall prevalence of prostate cancer in the Pakistani community. As Pakistan is a developing country, Pakistan's healthcare system faces several challenges [14,15], such as insufficient funding, inadequate infrastructure, a shortage of healthcare professionals, and inequitable distribution of resources. This study aims to systematically find, quantify, and summarise the available data on the prevalence of prostate cancer in Pakistan. This study aims to bridge this knowledge gap and provide valuable information for policymakers and physicians to plan better and prevent prostate cancer in Pakistan.

## 2 Methods

The PRISMA guidelines (attached in supplementary file S1) were followed for this systematic review and meta-analysis [16], and the protocol is included in the supplemental file in Table 1. In November 2022, it was registered with PROSPERO (with registration no. CRD42022376061).

**Table 1:** General characteristics of the included articles.

Author	Year	Study Design	Prevalence	Sample	Cases	Setting	Province	Sex	Working year	Male	Average Age	Risk of bias
Ikram et al. [25]	2023	Cross-sectional	5.8	124,795	7325	Both	Overall	Male	2015–2019	100	NA	Low
Ali et al. [26]	2023	Cross-sectional	3.1	8107	248	Urban	Sindh	Male	2015–2021	100	50.41	Low
Badar et al. [27]	2021	Cross-sectional	8.7	1324	115	Both	Punjab	Male	2017–2019	100	55	Medium
Ahmad et al. [28]	2016	Cross-sectional	4.8	4847	237	Both	Overall	Male	2014	100	40.5	Low
Qureshi et al. [29]	2016	Cross-sectional	2.2	5665	127	Both	Sindh	Male	2010–2015	100	60	Medium
Badar et al. [30]	2016	Cross-sectional	7.8	6771	526	Urban	Punjab	Male	2010–2012	100	40.2	Medium
Badar et al. [31]	2015	Retrospective	8.2	14,893	1222	Urban	Punjab	Male	1994–2012	100	67.12	Medium
Ahmad et al. [32]	2013	Retrospective	8.3	555	46	Urban	KPK	Male	2007–2012	100	42	Medium
Hanif et al. [33]	2009	Retrospective	4.2	7871	331	Both	Sindh	Male	2001–2008	100	61	Medium
Bhurgri et al. [34]	2002	Cross-sectional	9.4	7396	296	Urban	Sindh	Male	1998–1999	100	NA	Low
Bhurgri et al. [35]	2000	Cross-sectional	3.14	2160	68	Urban	Sindh	Male	1995–1997	100	65	Medium

**Search Strategy.** In this study, the PRISMA guidelines [16] were followed. To find information about the prevalence of prostate cancer in Pakistan, the following electronic databases were used: PubMed, Google Scholar, and Web of Science. We used a keyword procedure to match the search phrases to the Medical Subject Heading. Moreover, to combine and separate the search strings, boolean operators such as OR and AND were used. We used (prevalence) AND (prostate OR cancer OR prostate cancer OR PCa) AND (Pakistan OR Pakistani) to find essential articles. The prevalence of prostate cancer in Pakistan was mentioned in articles published between 2000 and 2023.

**Criteria for inclusion**. The study included peer-reviewed journal publications on the prevalence of prostate cancer in Pakistan. The articles were all original research publications in English that provided essential data on the study population, diagnostic processes, and the prevalence of prostate cancer in different locations around Pakistan.

**Criteria for Exclusion**. This study excluded articles that did not provide data on the prevalence of prostate cancer, had an unknown sample size, or had case-control studies.

**Data Extraction**. The authors of this study created a collaborative data extraction form in Microsoft Excel, which comprised the first author's name, year of publication, study design, sample size, overall prostate cancer prevalence, setting, province, gender, working year, and diagnosis method. To ensure accuracy and consistency, the reliability of the extracted data was thoroughly reviewed, and any inconsistencies were resolved through detailed discussion among the authors. Additionally, the eliminated data files were rigorously scrutinized for accuracy.

**Quality Assessment.** The JBI Critical Appraisal Checklist for Studies [17] was used by two authors (S.A. and F.H.) to assess the risk of bias in selected research independently. Each investigation was assigned a quality score between 0 and 9, which was used to determine the probability of bias. Studies with a score of 1–3 were considered to have a higher likelihood of bias, those with a score of 4–6 had a medium possibility of bias, and those with a score of 7–9 had a low chance of bias.

**Analysis.** We utilized the statistical software R (version 4.2.1) for all of our analyses, with a statistically significant P value of 0.05 being determined for the statistical pooling of prostate cancer prevalence in Pakistan. We employed Der Simonian-Laird models with random effects [18,19] and tested for statistical heterogeneity using the Cochrane Q-statistic and I^2^, where I^2^ greater than 50% defined heterogeneity [20,21]. We displayed the pooled results with 95% confidence intervals (CIs) and a forest plot. We computed the prediction interval to find the range in which the pooled effect deviates from the mean. Initially, we conducted visual and statistical analyses of publication bias using funnel plots and the Egger regression and Beggs tests [22,23]. Subgroup analysis was utilized in cases of significant heterogeneity to identify potential sources of heterogeneity. Various extracted variables, such as species (province, setting, and time), were used for subgroup meta-analyses.

Furthermore, meta-regression analysis was performed to explore heterogeneity further and determine the relationship between prostate cancer prevalence and study characteristics. The meta-regression included publication year, sample size, and investigation year. To better understand the impact of each study on the overall prevalence estimates, sensitivity analyses were conducted by systematically removing each study and re-analyzing the data. This helped to identify any potential outliers or sources of bias that may be driving the overall estimates. Furthermore, to assess the degree of agreement between the investigators, the Kappa statistic [24] was used to measure inter-rater reliability.

## 3 Result

**Fig. 1:**
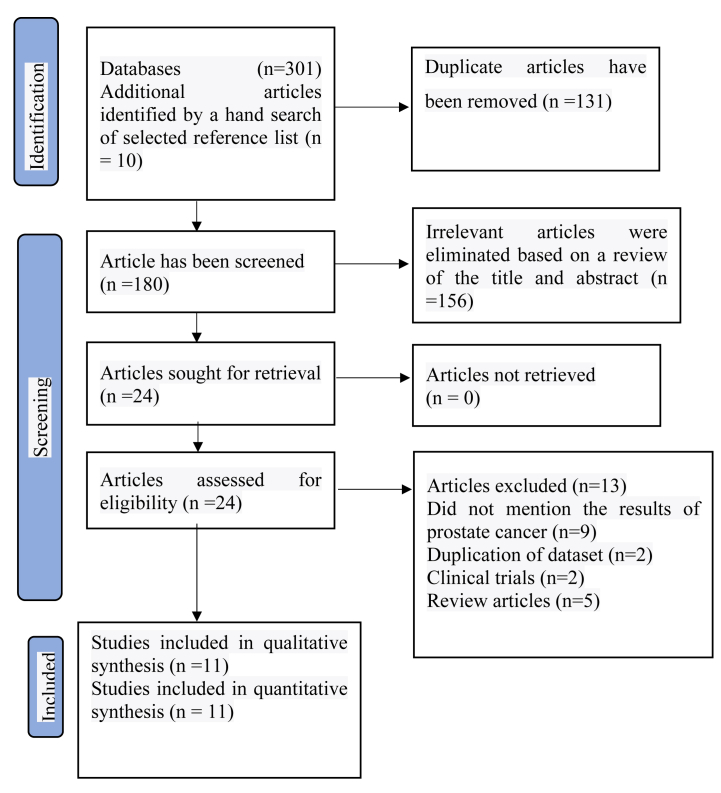
PRIMSA flow diagram^16^.

Fig. 1 depicts the adherence to the PRISMA guidelines for article selection, with 301 studies identified– 301 from database searches and ten from reference lists. Following deduplication (n = 131) and careful screening of the titles and abstracts of 180 articles, 156 studies were removed. The full text of the remaining 24 studies was examined to assess their eligibility for inclusion, and those that did not meet the inclusion criteria were excluded. Ultimately, eleven articles were selected for analysis. The inter-rater agreement for study selection was deemed good (Kappa = 0.79, p = 0.01).

### 3.1 Study characteristics

Table 1 outlines the general characteristics of the studies examined in this meta-analysis. The selected studies encompassed 184,384 individuals, with sample sizes varying from 555 [32] to 124,795 [25] individuals and a median of 6771 people. Of the total number of cancer cases, 10,541 cases were prostate cancer. The average age range of prostate cancer patients was reported in nine studies [[26], [27], [28], [29], [30], [31], [32], [33],^35^]. The articles considered in this meta-analysis were published between 2000 and 2023 and conducted between 1994 and 2021. Among them, three studies were retrospective [[31], [32], [33]], and eight used a cross-sectional design [[25], [26], [27], [28], [29], [30]]. The studies were conducted in three provinces of Pakistan, with three in Punjab [27,30,31], five in Sindh [26,29,[33], [34], [35]], one in Khyber Pakhtunkhwa [32], and two at the state level [25,28]. Five studies were performed in rural and urban areas [25,[27], [28], [29],^33^], and six were conducted only in urban [26,[30], [31], [32],^34^,^35^] areas. Regarding methodological quality, seven studies were found to have a moderate risk of bias [27,[29], [30], [31], [32], [33],^35^], while four studies had a low risk of bias [25,26,28,34]. None of the studies were determined to have a high risk of bias. The authors' inter-rater agreement for study selection and data extraction were 0.79 and 0.84, respectively.

### 3.2 Pooled prevalence

**Fig. 2:**
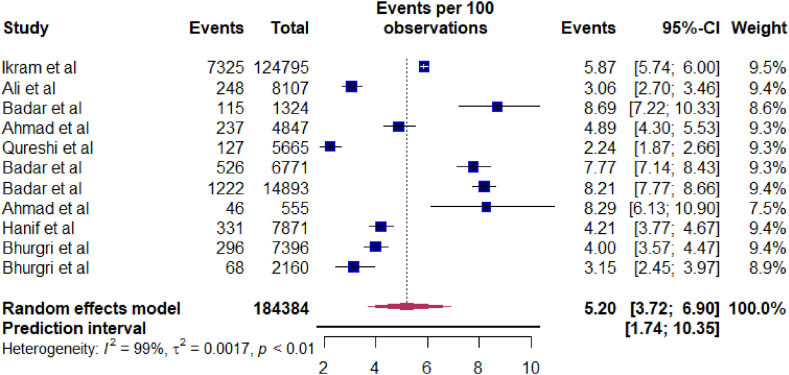
Forest plot of the pooled prevalence of prostate cancer in Pakistan.

**Fig. 3:**
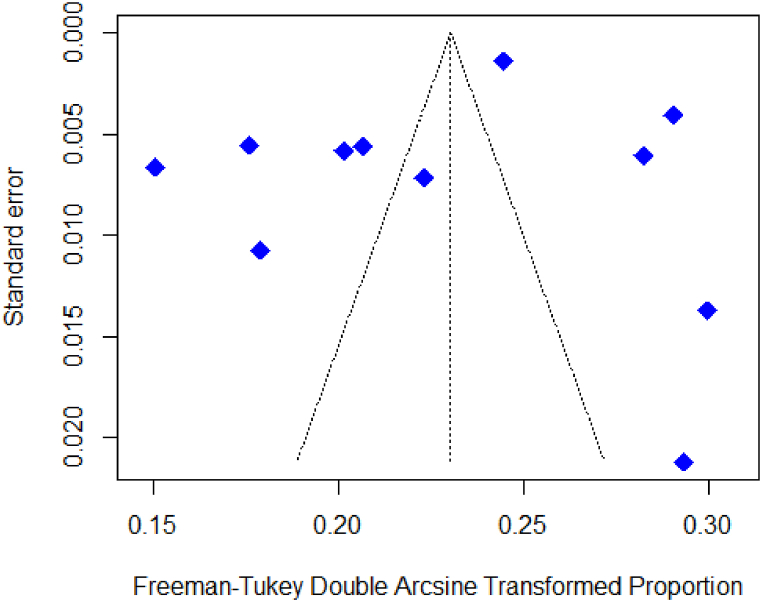
Funnel plot of prostate cancer in Pakistan.

**Fig. 4:**
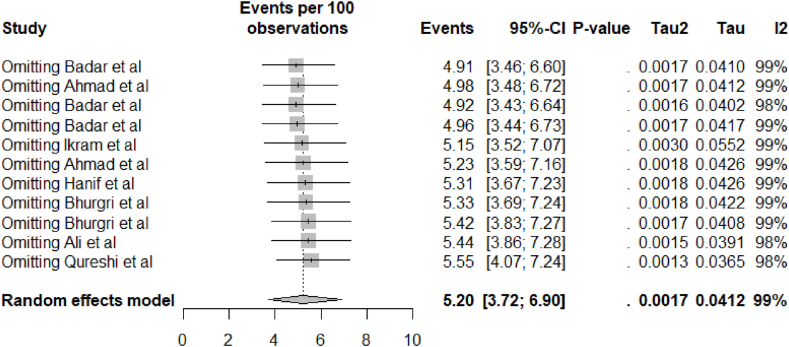
Sensitivity analysis of the prevalence of prostate cancer in Pakistan.

Table 2 describes the results of overall and subgroup meta-analyses of prostate cancer patients in Pakistan. The included studies reported a range of prevalence estimates for prostate cancer in Pakistan, from 2.2% to 9.4%. The random-effects total pooled estimated prevalence was 5.2%, with a wide range of 95% prediction intervals ranged from 1.74% to 10.35% (Fig. 2). The studies revealed significant heterogeneity among them (I^2^ = 0.99 and P < 0.01). The results from the funnel plot analysis (Fig. 3), Begg-Mazumdar rank test, and Egger's test indicate no publication bias in the meta-analysis. Furthermore, the sensitivity analyses revealed that removing any single study did not significantly impact on the prostate cancer's pooled prevalence, ranging from 4.91% to 5.55% (Fig. 4).

**Table 2:** Meta-analysis estimates of prostate cancer in Pakistan.

Variable	No. of Articles	No. of Participants	No. of Cases	Prevalence (95% CI)	I^2^, %	95%, Prediction interval	P-Value			
							Q test	Egger test	Begg Test	Subgroup Difference
Prostate Cancer	11	184,384	10,541	5.2 (3.72–6.90)	98.5	1.74–10.35	<0.001	0.4147	0.9397	
By Location										0.001
Punjab	3	22,988	1863	8.09 (7.36–8.86)	0	05.96–10.53	0.39			
Sindh	5	31,199	1070	3.3 (2.37–4.38)	92.4	1.07–06.69	<0.001			
KP	1	555	46	8.29 (6.13–10.74)						
By Setting										0.7092
Urban	6	39,882	2406	5.47 (3.05–8.52)	98.8	0.35–16.02	<0.001			
Both	5	144,502	8135	4.92 (2.44–8.20)	98.4	0.61–12.99	<0.001			
Survey duration										0.0374
≤2010	3	17,427	695	3.88 (2.72–5.23]	61.6	0.35–10.95	0.07			
>2010	8	166,957	9846	5.8 (3.76–8.24)	98.7	1.67–12.19	<0.001

### 3.3 Subgroup analysis

A subgroup meta-analysis was performed to detect the significant sources of statistical heterogeneity based on geographical location, setting, and survey duration. Subgroup meta-analysis revealed that the prevalence of prostate cancer was highest in Khyber Pakhtunkhwa province (8.29%; 95% CI: 6.13–10.74%, n = 1), followed by Punjab (8.09%; 95% CI: 7.36–8.86%, n = 3), and lowest in Sindh (3.30%; 95% CI: 2.37–4.38%; n = 5). Pooled prevalence estimates for prostate cancer were 3.88% (95% CI: 2.72–5.23%; n = 3) from 2000 to 2010 and 5.80% (95% CI: 3.76–8.24%; n = 8) from 2011 to 2023. In recent years, prostate cancer prevalence has increased significantly. When stratified by setting, the pooled prevalence of prostate cancer was highest in urban (5.47%; 95% CI: 3.05–8.52%, n = 6) than in both (rural and urban) (4.92%; 95% CI: 2.44–8.2%, n = 5). Based on univariate meta-regression analysis, no statistically significant relationship existed between prevalence and publication year, year of research, age and study design (Table 3).

**Table 3:** Univariate meta-regression analysis of prostate cancer.

Variable	Beta (β)	p-value	95% CI	R^2^%
Publication Year	−0.207	0.5647	−0.0992–0.0577	0.0
Year of Research	0.0011	0.6459	−0.0041–0.0062	0.0
Age	0.2826	0.0692	−0.1090–0.6742	46.11
Study design	0.0426	0.2619	−0.0379–0.1232	0.0

## 4 Discussion

Prostate cancer is a significant health concern in Pakistan. According to the Pakistan National Cancer Registry, prostate cancer is the second most common cancer among men in the country [36]. Therefore, we performed this systematic review and meta-analysis to provide a pooled estimate of prostate cancer in Pakistan. This meta-analysis is based on data published between 2000 and 2023. The study utilized data from 11 distinct data sets with 184,384 prostate cancers from geographically varied Pakistani populations. Our study aims to give helpful information about prostate cancer to the health authorities of Pakistan so that something can be done to prevent deadly diseases like prostate cancer. The overall prevalence of prostate cancer was 5.20%, which shows the prevalence of prostate cancer in Pakistan. The findings of this meta-analysis are almost consistent with the study conducted in Iran, at 6.3% [37] and lower than in the studies conducted in Nigeria, at 8.8% [38]. This disparity may be attributable to variations in research techniques, sample size, social, environmental, and genetic factors, and the universal definition of prostate cancer.

Subgroup meta-analysis by study location revealed that the pooled prevalence of prostate cancer was highest in Khyber Pakhtunkhwa (8.29%), followed by Punjab (8.09%), and lowest in Sindh (3.3%). The disparity in the prevalence of prostate cancer in different provinces may be due to socioeconomic and sociocultural differences between populations, variations in screening methods, or there may be no higher research on prostate cancer in those areas. Subgroup meta-analysis by setting revealed that the prevalence of prostate cancer was significantly higher in Urban areas than in Rural areas. This study also found that the prevalence of prostate cancer has increased over time, with the prevalence being 3.88% from 2000 to 2010, while the prevalence from 2011 to 2023 was 5.80%.

The following are some of the limitations of our study: seven studies had a medium risk of bias, while four had a low risk of bias. Second, the results are based on data from only three provinces. They lack data from two populous regions, Baluchistan and Azad Kashmir, limiting generalization to the entire country. Third, it is limited to peer-reviewed studies, which may result in publication bias. Additionally, high heterogeneity was detected in the analysis, which is common in meta-analyses of prevalence data. Finally, our study only included peer-reviewed studies and excluded grey literature. In this study, despite limitations, our transparent approach involved publishing a study protocol and utilizing scientific and statistical methods, including subgroup and meta-regression analyses, to account for potential influencing variables in our estimate.

## 5 Conclusion

This study examines the prevalence of prostate cancer in the male Pakistani population from 2000 to 2023. According to this study, the pooled prostate cancer prevalence in Pakistan was 5.20%. However, it differed significantly by geographical subgroup, with Khyber Pakhtunkhwa having the highest rate and Sindh having the lowest. A nationwide cancer registry must be established.

## Ethical statement

The ethical statement is not applicable in this study as this is a review paper, and we are using secondary published information.

## Author contribution statement

Sohail Akhtar: Conceived and designed the experiments; Analyzed and interpreted the data; Contributed reagents, materials, analysis tools or data; Wrote the paper.

Fazal Hassan; Sadique Ahmad; Mohammed A El-Affendi; Muhammad Imran Khan: Analyzed and interpreted the data; Contributed reagents, materials, analysis tools or data; Wrote the paper.

## Data availability statement

Data included in article/supplementary material/referenced in article.

## Declaration of competing interest

The authors declare that they have no known competing financial interests or personal relationships that could have appeared to influence the work reported in this paper.
